# Keratinocytes regulate intraepithelial lymphocytes homing and mediate mucosal barrier integrity via JAK2/STAT3 signaling in oral lichen planus

**DOI:** 10.3389/fimmu.2026.1794867

**Published:** 2026-03-30

**Authors:** Dong-Yang Zhou, Fang Wang, Chao-Fan Bao, Gang Zhou

**Affiliations:** 1 State Key Laboratory of Oral & Maxillofacial Reconstruction and Regeneration, Key Laboratory of Oral Biomedicine Ministry of Education, Hubei Key Laboratory of Stomatology, School & Hospital of Stomatology, Wuhan University, Wuhan, China; 2 Department of Oral Medicine, School and Hospital of Stomatology, Wuhan University, Wuhan, China

**Keywords:** intraepithelial lymphocytes, JAK/STAT, lymphocyte homing, oral lichen planus, oral mucosal barrier

## Abstract

**Introduction:**

Oral lichen planus (OLP) is a common T-cell-mediated inflammatory disease affecting the oral mucosa. Intraepithelial lymphocytes (IELs), a unique subset of T cells, play a crucial role in regulating mucosal immune responses. However, the mechanisms by which keratinocytes (KCs) regulate the homing migration of OLP IELs and their involvement in mucosal barrier disruption remain unclear.

**Methods:**

This study conducted colocalization and quantitative analysis of the expression of E-cadherin, CD103, CD8α, ZO-1, and Occludin. A three-dimensional simulation homing model of oral mucosal tissue was constructed. Short hairpin RNAs (shRNAs) were designed to inhibit E-cadherin and CD103 of KCs and OLP IELs. The JAK/STAT pathway was inhibited using AG490 and ruxolitinib (RPM). The expression levels of ZO-1 and occludin were detected.

**Results:**

CD8α and CD103 were highly expressed in OLP, while the expression of E-cadherin, ZO-1, and Occludin was decreased. Silencing KCs' E-cadherin and IELs' CD103 significantly inhibited the homing migration of OLP IELs. After inhibiting the JAK/STAT pathway, KCs proliferation was reduced, while Bax and caspase-3 expression were upregulated and Bcl-2 expression was downregulated. The homing migration of OLP IELs was inhibited, with decreased expression of p-JAK2/JAK2 and p-STAT3/STAT3. Furthermore, ZO-1 and Occludin were upregulated.

**Discussion:**

The regulation of KCs on homing migration of OLP IELs depended on KCs' E-cadherin and IELs' CD103. By downregulating JAK2/STAT3 phosphorylation, KCs proliferation was inhibited and apoptosis was induced, which has therapeutic benefits for OLP epithelial dysplasia. Meanwhile, upregulation of mucosal barrier molecule expression helps maintain the integrity of the mucosal barrier.

## Introduction

Oral lichen planus (OLP) is a common oral mucosal disorder that predominantly affects middle-aged women (1). The Andreasen Classification categorizes OLP into six types: reticular, patchy, atrophic, vesicular, erosive, and papular (2). OLP typically follows a prolonged course, and a minority of cases may progress to malignancy, classifying it as oral potential malignant disorders (3). The pathogenesis of OLP is not fully understood but is generally considered a chronic inflammatory disease mediated by T lymphocytes and associated with immune abnormalities (4, 5). The histopathological features of OLP include liquefaction degeneration of basal cells and banded chronic inflammatory infiltration mainly composed of superficial lymphocytes in the lamina propria (6, 7). The core mechanism involves antigen presentation by oral mucosal keratinocytes (KCs), triggering cell-mediated immune responses and treating these cells as antigen-specific CD8^+^T cells (4, 8). Studies have demonstrated that the liquefaction degeneration of epithelial basal cells in OLP represents a classic epithelial-mesenchymal transition (EMT). The downregulation of epithelial cadherin (E-cadherin), an epithelial cell marker, is recognized as a hallmark of EMT (9).

Intraepithelial lymphocytes (IELs) are immune cells within the body immune system that maintain the closest contact with foreign antigens and microorganisms, serving as the first responders to initiate immune responses. Our previous research indicated that CD8α expression and CD8αα^+^ cells were upregulated in the epithelium of OLP, and CD4^+^CD8α^+^ IELs may be the main phenotype of IELs in OLP (10). Excessive lymphocyte homing is a key mechanism contributing to mucosal immune imbalance and barrier damage, with integrins playing a critical role during homing process (11, 12). Integrins are heterodimers composed of two subunits (α and β), expressed on the surface of most cells, and mediate bidirectional signaling between the intracellular and extracellular environments (13). The interaction between E-cadherin and integrin alpha-E (CD103) not only facilitates mechanical sensing but also transduces extracellular and intracellular mechanical signals to regulate myosin connections, thereby modulating the strength of adhesion junctions (14). CD103-deficient mice exhibit a reduction in IELs due to impaired retention within the epithelial cell compartment, suggesting that CD103 primarily functions as a retention receptor (15, 16). Studies have demonstrated that IELs, together with intestinal epithelial cells, form both the mechanical and immune barriers of the intestine. Therefore, the proper function of IELs directly influences the integrity of the mucosal immune barrier (17). However, the relationship between mucosal base layer damage in OLP and IELs remains unexplored.

The Janus kinase/signal transducer and activator of transcription (JAK/STAT) pathway serves as a critical hub for cytokine signal transduction, playing a central role in regulating cell proliferation, differentiation, apoptosis, and immune responses (18–20). The classical activation of this pathway involves cytokine binding to receptors, which activates JAK kinases and subsequently phosphorylates STAT proteins. The phosphorylated STAT proteins (p-STAT) form dimers and translocate to the nucleus, where they function as transcription factors to modulate the expression of downstream target genes. While the exact pathogenesis of OLP is unclear, current understanding suggests that T lymphocytes activate the JAK pathway, eventually triggering the release of cytokines, including interferon-gamma (4). Studies have shown JAK1 and JAK3 overexpression in OLP lesions, further supporting the role of JAK signaling in its pathogenesis (21). A recent systematic review supports involvement of JAK/STAT-mediated immune signaling in the pathogenesis of lichen planus, which provides a mechanistic rationale for JAK inhibition in refractory OLP (22). Currently, the JAK1/3 inhibitor tofacitinib has been successfully used to treat OLP, cutaneous lichen planus, and lichen planus of the penis (LPP) (23, 24). Baricitinib, due to its ability to interfere with IFN-γ signaling through the JAK2/STAT1 pathway, is also a potential therapeutic candidate for OLP.

JAK2, a member of the JAK family, is a non-receptor tyrosine kinase that plays a key role in cytokine signal transduction. Abnormal activation of JAK2 is associated with various immune system disorders (25). JAK2 participates in cytokine signaling, which plays a crucial regulatory role in immune responses. However, research on whether JAK2 is involved in the immunopathological mechanisms of OLP remains limited.

Based on the above, this study explored the roles of E-cadherin and CD103 in OLP IELs migration by KCs. By combining AG490 and RPM, the effects of dual blockade of the JAK/STAT signaling pathway on KCs and the potential molecular regulatory mechanisms for OLP IELs homing migration were analyzed.

## Materials and methods

### Patients and samples

Tissue samples from patients with OLP and healthy controls were collected; all participants were enrolled from the Department of Oral Medicine, School and Hospital of Stomatology, Wuhan University. OLP was diagnosed according to the World Health Organization (WHO) criteria (7), based on clinical and histological examination results. The screening criteria for OLP patients were based on our previously published inclusion and exclusion criteria (26). All patients provided informed consent prior to tissue sampling. The study was approved by the Ethics Committee Board of the School and Hospital of Stomatology, Wuhan University (No.: 2022A29) and was conducted in accordance with the principles of the Declaration of Helsinki. The clinical characteristics of the tissue samples are detailed in **Table 1**.

**Table 1:** Clinical characteristics of OLP patients and controls.

Clinical characteristics	OLP	Control
Total number	16	10
Gender		
Male	7	4
Female	9	6
Age (years)		
Range	21-65	23-69
Mean ± SD	43.75 ± 9.28	36.33 ± 13.65
Type		
Non-erosive	11	
Erosive	5	
Medication use	None	None

OLP, Oral lichen planus.

### Immunohistochemistry

The OLP tissue slices embedded in paraffin were dried, deparaffinized, and rehydrated. Subsequently, the slices were placed in EDTA buffer (1 mM, pH 9.0) or citrate buffer (10 mM, pH 6.0) for microwave antigen retrieval. After treatment with hydrogen peroxide and blocking with normal goat serum, the sections were incubated overnight at 4 °C with primary antibodies: mouse anti-CD8α monoclonal antibody (1:200, Cat # GB12068, Servicebio, China), mouse anti-E-cadherin monoclonal antibody (1:100, Cat # GB12083, Servicebio, China), mouse anti-CD103 monoclonal antibody (1:500, Cat # 14-1038-82, eBioscience, China), mouse anti-ZO-1 mAb (1:200; CST, USA) and Occludin mAb (1:200; CST, USA). The slices were then incubated with HRP polymer-conjugated anti-mouse secondary antibody for 10 min at 37 °C and visualized using diaminobenzidine (DAB) solution. Hematoxylin was used to counterstain the nuclei. Images were acquired using CaseViewer (3D HISTECH Ltd., Budapest, Hungary). Immunohistochemistry staining images were analyzed by selecting five randomly chosen, non-overlapping areas at 400× magnification. The mean optical density (MOD) was used to quantify positive staining using Image-Pro Plus version 6.0 (IPP, Media Cybernetics).

### Immunofluorescence

The thickness of the formalin-fixed, paraffin-embedded tissue sections was 4 µm. The sections were dewaxed in xylene and rehydrated through a graded alcohol series. Antigen retrieval was performed by microwave treatment for 20 min in EDTA buffer (1 mM, pH 9.0). Subsequently, the sections were blocked with 1% bovine serum albumin (BSA) (Cat # 4240GR005, BioFroxx, Germany) and incubated with mouse anti-CD8α mAb (1:200, Cat # GB12068, Servicebio, China), mouse anti-E cadherin mAb (1:100, Cat # GB12083, Servicebio, China), and mouse anti-CD103 mAb (1:500, Cat # 14-1038-82, eBioscience, China). Staining was performed using the Think Color-4 ColorMou Multi-Label Kit (NFB, China).

The actions were blocked with 1% BSA (Cat # 4240GR005, BioFroxx, Germany) and double-stained with rabbit anti-ZO-1 mAb (1:200, Cat # ab307799, Abcam, USA) and rabbit anti-Occludin mAb (1:200; Cat # ab224202, Abcam, USA). Next, the sections were labeled with Dylight 594-conjugated goat anti-rabbit IgG (1:200, Cat # A23420, Abbkine, China). The nuclei were counterstained with DAPI.

The sections were observed and photographed under a fluorescence microscope (Leica, Solms, Germany). Images were analyzed using ImageJ software (National Institutes of Health, Bethesda, Maryland, USA).

### Separation and culture of OLP IELs

OLP tissue was immersed in 0.25% Dispase II (Cat # 40104ES60, Yeasen, China) for 16 h, then rinsed with DPBS. The epithelial layer was separated using ophthalmic forceps and placed in DMEM medium supplemented with 4% FBS, 5 mM EDTA (Cat # G1105-500ML, Servicebio, China) and 0.145 mg/mL disulfide (GC205010-1g, Servicebio, China) at 37 °C for 40 min under mechanical shaking. After centrifugation, the supernatant was discarded, and the cells were washed with DMEM containing 2 mM EDTA, followed by filtration through a 70 μm cell strainer and centrifugation. Cells were resuspended in RPMI-1640 containing 5% FBS (Cat # 110118611, EveryGreen, Hangzhou, China) and subjected to gradient centrifugation with Percoll/PBS (Percoll, Cat # P8370, Solarbio, China) at 30%, 55%, and 70% for purification. A small portion of the cells was suspended in PBS for trypan blue staining and counting, while the remaining cells were reserved for subsequent experiments.

Anti-human CD3 ϵ/CD3E (Cat # IT-300-CD3E-m1, Suzhou, China) was placed on a 96-well round-bottom plate (1 μg/mL, 50 μL per well) and incubated at 37 °C for 2 hours. Then the plate was washed three times with sterile PBS. IELs at 5×10^5^/ml were resuspended in the 96-well plate and the following cytokines was added: IL-2 (10 IU/mL), IL-3 (100 IU/mL), IL-4 (100 IU/mL), and soluble IL-15 (100 ng/mL). 200 μL system was obtained and cultured at 37 °C in a carbon dioxide incubator for 48 hours. The cells were washed twice and resuspended at 1×10^5^ per well in a medium (RPMI1640 + 10% FBS) containing IL-2 (10 IU/mL). The fresh medium containing IL-2 was replaced every 3–4 days.

### Lentiviral transfection

Three types of recombinant lentiviruses were used to manipulate the expression of the E-cadherin and CD103 genes. Specifically, sh-E-cadherin lentiviruses were employed to knock down E-cadherin expression, with sh-NC, containing empty vectors, serving as negative controls. The sh-CD103 lentiviruses were employed to knock down CD103 expression, with sh-NC, containing empty vectors, serving as negative controls. Lentivirus samples were obtained from GenePharma (Suzhou, China). After 6–8 h of lentivirus cultivation, the cells were chosen with 2 µg/mL puromycin.

### Quantitative real-time PCR

Lentiviral transfection efficiency was determined by quantitative real-time PCR (qRT-PCR). Total RNA was extracted using TRIzol (AXYGEN, NY, USA), and reverse transcription was performed using the HiScript III RT SuperMix for qPCR Kit (Vazyme, Nanjing, China). Then, qRT-PCR was conducted using the ChamQ Universal SYBR qPCR Master Mix (Vazyme) on the CFX Connect™ Real-Time System (Bio-Rad Laboratories, Hercules, CA, USA). Gene expression changes were determined using the comparative 2^−ΔΔCt^ method. Each experiment was performed in triplicate. The primers used were as follows: GAPDH, F-5′-TGGGTTTCCCGTTGATGA-3′ R-5′-AGGGCTGCCTTCTCTTGT-3′; E-cadherin, F-5′-TCATGAGTGTCCCCCGGTAT-3′, R-5′-TCTTGAAGCGATTGCCCCAT-3′; CD103, F-5′-CAAGAGGTCATCTGCTCATGT-3′, R-5′-GGACAGAACTGCAACGAAGC-3′.

Similarly, the relative expression levels of ZO-1 and Occludin were calculated using the 2^−ΔΔCt^ method. The primers used were as follows: GAPDH, F-5′-TGGGTTTCCCGTTGATGA-3′ R-5′-AGGGCTGCCTTCTCTTGT-3′, ZO-1, F-5′-ATTCAGGTCGCTCGCATGAC-3′, R-5′-ACTGCGTGGAATGATCGGAG-3′ Occludin, F-5′-CTCGGTACAGCAGCAATGGT-3′, R-5′-TCATAGTGGTCAGGGTCCGT-3′. All primers used in this study were synthesized by Sangon™ Biotech (Shanghai, China).

### Ethynyl-2’-deoxyuridine assay

EdU assays was monitored by a BeyoClick EdU Cell Proliferation Kit with DAB (Beyotime, China). The cells were incubated for ~2 h with 10 µM EdU, immobilized in 4% PFA for ~15 min, infiltrated in PBS including 0.3% Triton X-100, and then dyed with Click Additive Solution at 37 °C for 1 h. Cell nuclei were colored by DAPI (Beyotime). Pictures were caught through an EVOS M5000 Fluorescence Microscope (Thermo Fisher Scientific, USA).

### CCK-8 assay

Cells were seeded into 96-well plates at a density of 2 × 10^5^ cells per well. Cell proliferation was measured using the CCK-8 assay (Dojindo, Japan) by recording the OD450 value during the final 2 hours of culture on days 1, 2, 3, and 4.

### Transwell assay

To evaluate the migratory and invasive potential of KC-sh-NC, KC-sh-E-cadherin, IEL-sh-NC, and IEL-sh-CD103 cells, a 24-well transwell system from corning with an 8-µm pore size was used. In the transwell migration assay, the lower chamber was filled with medium containing 10% FBS, while the upper chamber was seeded with 1 × 10^5^ pre-treated cells in medium also containing 10% FBS. For the invasion assay, the upper transwell chambers were coated with 100 µL of Matrigel (ABW; 082704, Shanghai, China) to create a continuous membrane. In this setup, the upper chamber was inoculated with 5 × 10^5^ cells in serum-free medium, and the lower chamber contained medium supplemented with 20% FBS. After 24 hours of incubation, the upper chambers, housing cells that had migrated or invaded to the lower surface of the membrane, were fixed and stained with 0.5% crystal violet. Remaining cells on the upper surface were removed using a cotton swab. Random fields were photographed under a microscope and quantified using ImageJ software (National Institutes of Health, Bethesda, Maryland, USA).

### Hoechst/PI staining

KC-sh-NC, KC-sh-E-cadherin, IEL-sh-NC, and IEL-sh-CD103 cells were stained with the DNA-specific fluorescent dyes Hoechst 33342 and propidium iodide (PI). Cells with nuclei stained by Hoechst 33342 were considered total cells, while those stained with PI were classified as dead cells. Staining was analyzed using a phase-contrast inverted microscope (Soptop ICX41, Ningbo, China). Cell death was quantified as the ratio of PI-positive cells to Hoechst-positive cells.

### Terminal deoxynucleotidyl transferase dUTP Nick-end labeling assay

Cells were harvested and fixed with 4% paraformaldehyde for 1 h at room temperature (RT), then dehydrated in ethanol and embedded in paraffin using conventional protocols. Four-micrometer sections from each paraffin-embedded cell aggregate, deparaffinized in xylene, and hydrated in rehydrated water. Sections were analyzed following the protocol (Roche, Germany). Briefly, antigen retrieval was carried out by incubating sections with Proteinase K (Dako, USA) for 10 min at RT. Sections were then incubated in blocking solution (3% Bovine Serum Albumin in 10% goat serum) for 15 min, washed twice with PBS, and incubated for 1 h at 37 °C in the dark with a dUTP nick-end labeling (TUNEL) reaction mixture for *in situ* detection of cell death. After washing three times with PBS, cells were incubated at RT with the converted POD solution for 1 h. After rinsing with PBS for three times, 50 μl of DAB substrate was added. Following incubation for 2 min under RT, samples were counterstained with hematoxylin and analyzed by light microscopy. Then, apoptosis was expressed as percentage of TUNEL-positive nuclei in five random independent HPFs (400×). Experiments were repeated for three times.

### 3D simulation homing model of oral mucosal tissue

Human gingival fibroblasts (GFs) were seeded at a density of 1.2 × 10^6^ cells per well onto a 12-well transwell membrane with 0.4 μm pores and incubated at 37 °C for 2 h. The chambers were then immersed in DMEM/F-12 medium (Gibco, Grand Island, NY, USA) for 2 days. Human KCs were seeded at a density of 0.6 × 10^6^ cells per well onto the surface layer and cultured at 37 °C in a 5% CO_2_ incubator for 5 days, with the culture medium changed every 2 days. After 5 days, the complete KC culture medium (K-SFM, Cat # 17005-042, Gibco, USA) in the transwell upper chamber was removed, and fresh complete DMEM medium (Gibco) was added to the lower chamber to match the height of the tissue model in the upper chamber. The model was then further cultured for 10 days to develop a full-thickness mucous membrane. Cells were seeded into 96-well plates at a density of 2 × 10^5^ cells per well. Cell proliferation was measured using the CCK-8 assay (Dojindo, Japan) by recording the OD450 value during the final 2 h of culture on days 1, 3, and 5.

On day 10, IELs were seeded onto the transwell upper chamber at a density of 5×10^5^ cells/500 μL per well. Three distinct 3D models were developed based on cellular components: the KC group (KC), the KC and GF group (KC/GF), and the KC, GF, and IEL group (KC/GF/IEL). KCs in each group were stimulated with 10 μg/mL lipopolysaccharide (LPS) for 24 h, and resistance values were measured using the RE1600 transmembrane electrical resistance instrument.

The models were divided into four groups based on the addition of inhibitors: (1) Control group (treated with K-SFM medium containing an equal concentration of PBS); (2) AG490 group (treated with K-SFM medium containing 50 μmol/L AG490) (AG490, Cat # HY-12003, MCE); (3) RPM group (treated with K-SFM medium containing 20 nmol/L RPM) (RPM, Cat # HY-10219, MCE); and (4) AG490+RPM group (treated with K-SFM medium containing 50 μmol/L AG490 and 20 nmol/L RPM). The control group served as the baseline; the AG490 and RPM groups were used to assess the blocking effects of the JAK2 and STAT3 pathways, respectively. The AG490+RPM group was used to investigate whether dual inhibition had a synergistic effect. The four groups were incubated at 37 °C in 5% CO_2_ for 5 days, with the medium changed every 2 days. After 5 days, K-SFM in the transwell upper chamber was removed, and fresh DMEM complete culture medium was added to the lower chamber, with the medium height matching that of the tissue model in the upper chamber. The models were cultured continuously for 10 days to form full-thickness mucosal tissue. The transwell membrane was then cut, and single-cell suspensions were collected by enzymatic digestion. All experiments were independently repeated three times.

### MTT assay

Cell proliferation was assessed using the MTT assay with the EZcount™ MTT Cell Assay Kit, following the manufacturer’s instructions. Briefly, KCs were seeded in 96-well plates at an appropriate density and allowed to adhere overnight. The cells were then cultured in four different media (Control: K-SFM medium containing an equal concentration of PBS; AG490: K-SFM medium containing 50 μmol/L AG490; RPM: K-SFM medium containing 20 nmol/L RPM; AG490+RPM: K-SFM medium containing 50 μmol/L AG490 and 20 nmol/L RPM) and incubated for 24 hours under standard conditions. After treatment, MTT reagent was added to each well and incubated for 4 hours to allow for formazan formation. The resulting crystals were dissolved using a solubilizing buffer, and absorbance was measured at 450 nm using a microplate reader.

### Homing migration

KCs in 3D simulation homing models of oral mucosal tissue were replaced with RFP-labeled E-cadherin-positive (E-cadherin^+^) and E-cadherin-negative (E-cadherin^-^) KCs, respectively. GFP-labeled CD103-positive (CD103^+^) and CD103-negative (CD103^-^) IELs were introduced into the homing models. Four homing models were established and designated as E-cadherin^+^CD103^+^, E-cadherin^+^CD103^-^, E-cadherin^-^CD103^+^, and E-cadherin^-^CD103^-^. Live cell migration within the models was observed at 0, 2, 5, and 7 days using a combination of laser confocal microscopy and differential interference contrast (DIC) imaging.

Confocal fluorescent microscopy was performed with a Leica SP5 (Leica, Wetzlar, 3D reconstruction of co-culture samples was created after imaging with 5 µm z-stacks spacing using a Zeiss Spinning Disc Axio Observer Z1 (Zeiss, Oberkochen, Germany).

The ZEISS Axio Observer Z1 (Zeiss, Oberkochen, Germany) live-cell imaging system was used. Co-cultures were seeded in 4-well ibidi plates and incubated at 37 °C with 5% CO_2_ during imaging. Images were acquired using a 25× objective lens. Models were imaged with differential interference contrast (DIC) illumination. RFP was excited with a 532 nm laser, and GFP with a 488 nm laser. Time-lapse experiments were conducted at 1-minute intervals. Bicolor confocal illumination was employed for imaging. IEL tracks were represented by yellow lines, while KC tracks were represented by blue lines. Migration speed was calculated by measuring the horizontal displacement (i.e., track length) of cells over time during live imaging. Maximum displacement was defined as the average of the first 15 displacement values in each time-lapse sequence. Overnight incubation was performed prior to imaging, and analysis was conducted under fixed field conditions using a 25× objective lens.

### ELISA assay

The detection of Bax, Bcl-2, and caspase-3 in the supernatant of cultured models was performed using ELISA kits (4A Biotech Co., Ltd., Beijing, China) following the manufacturers’ instructions. Proteins were extracted using M-PER mammalian protein extraction reagent (Thermo Scientific, Waltham, Massachusetts, USA), and protein quantification was performed using BCA assay kit (Beyotime, Nanjing, China). The expression levels of JAK2, p-JAK2, STAT3, and p-STAT3 proteins in cell lysates were detected using ELISA kits (Human JAK2 ELISA KIT, Mlbio, ml105594, China; Human Phosphorylated tyrosine kinase 2 P-JAK2 ELISA KIT, Mlbio, China; Human Signal Transducer And Activator Of Transcription 3 STAT3 ELISA kit, Human solubility Signal Transducer and Activator Of Transcription 3 p-STAT3 ELISA Kit, XIN YU BIOTECHNOLOGY, Shanghai, China).

### Western blot

Proteins were obtained from cells in 3D OMM by utilizing M-PER mammalian protein extraction reagent (Thermo Scientific, Waltham, MA, USA), and protein quantification was performed using a BCA kit (Beyotime, Nanjing, China). Afterward, 30 micrograms of protein from each group underwent electrophoresis using a 12% SDS-PAGE gel and were then transferred onto polyvinylidene difluoride (PVDF) membranes (Millipore, Billerica, MA, USA) using the wet transfer technique. After being blocked with 5% skimmed milk for 1 h, the membranes were incubated with primary antibodies overnight at 4°C. After incubating with secondary antibodies at room temperature the following day, the membranes were observed using the Odyssey LI-COR scanner after being treated with the SuperSignal™ West Femto Reagent (Thermo Scientific, Waltham, MA, USA).

### Co-immunoprecipitation

The cells in 3D OMM were placed in an immunoprecipitation lysis buffer (Cat # KGB5202-100, Jiangsu, China) containing 1% PMSF, 1% protease, and phosphatase inhibitors, and lysed on ice for 30 minutes. After centrifugation, the supernatant was collected and mixed with E-cadherin primary antibody (1:1000, Cat # GB12083, Servicebio, China) and Protein A/G immunoprecipitation magnetic beads (Cat # B23202, Shanghai, China). The mixture was then incubated overnight with slow shaking at 4 °C. The next day, the supernatant was removed from the mixture and the beads were washed. The interaction between E-cadherin and CD103 was detected using Western blotting.

### Statistical analysis

The data were analyzed using GraphPad Prism 8. The statistical analyzes yielded a summary of the mean ± standard deviation (SD) from at least three independent experiments. The normal distribution was determined using the Shapiro-Wilk test. To assess the disparities, the t-test for Student’s, along with the one-way ANOVA incorporating Bonferroni correction, was utilized. The meaning of significance was determined as * *p* < 0.05, ** *p* < 0.01, *** *p* < 0.001.

## Results

### CD8α and CD103 were highly expressed, E-cadherin was lowly expressed in OLP

**Figure 1:**
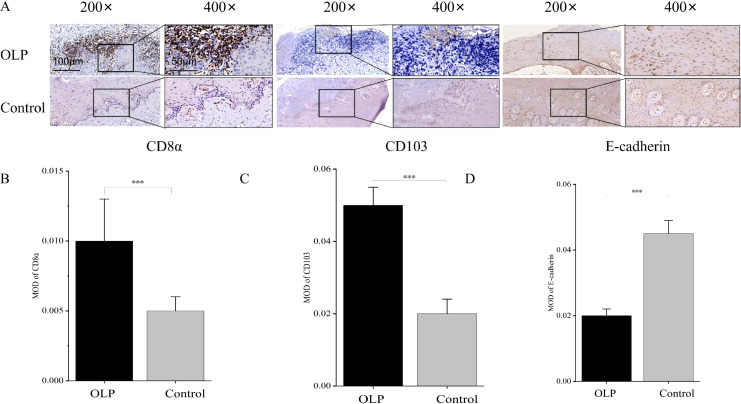
CD8α and CD103 were highly expressed, E-cadherin was lowly expressed in OLP. **(A)** CD8α, CD103, and E-cadherin were primarily localized in the spinous layer and the epithelial basal layer, with all three proteins predominantly localized to the cell membrane. **(B, C)** CD8α and CD103 expression were significantly increased in the epithelial tissues of OLP. **(D)** The expression of E-cadherin was significantly reduced in the epithelial tissues of OLP. *p < 0.05; **p < 0.01; ***p < 0.001. CD103, Integrin alpha-E; E-cadherin, Epithelial cadherin; OLP, Oral lichen planus; MOD, Mean optical density; CD8α, Cluster of differentiation 8α.

**Figure 2:**
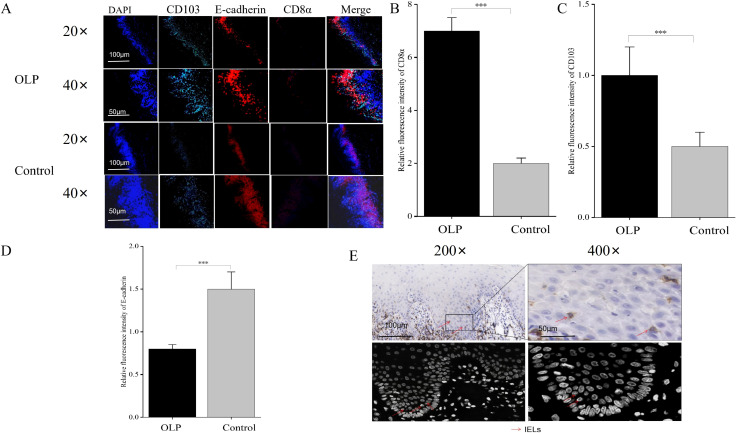
CD8α and CD103 were highly expressed, E-cadherin was lowly expressed in OLP. **(A–C)** The expression of CD8α and CD103 was significantly upregulated in OLP. **(D)** The expression of E-cadherin was significantly downregulated in OLP. **(E)** IELs were located in the intercellular space of epithelial cells, with tight junctions to the epithelial cells above and the basement membrane below. Red arrow: IELs. *p < 0.05; **p < 0.01; ***p < 0.001. CD103, Integrin alpha-E; E-cadherin, Epithelial cadherin; OLP, Oral lichen planus; IELs, Intraepithelial lymphocytes; CD8α, Cluster of differentiation 8α.

CD8α, CD103, and E-cadherin were primarily localized in the spinous and basal layers of the epithelium (**Figure 1A**), with all three proteins predominantly found on the cell membrane (**Figure 1A**). Quantitative MOD analysis revealed a significant increase in the expression of CD8α and CD103 in the epithelial tissues of OLP compared to control (*p* < 0.001) (**Figures 1B, C**). Conversely, E-cadherin expression was significantly decreased in the epithelial tissues of OLP (*p* < 0.001) (**Figure 1D**). Immunofluorescence staining confirmed the significant upregulation of CD8α and CD103 (*p* < 0.001) (**Figures 2A–C**) and the significant downregulation of E-cadherin (*p* < 0.001) (**Figure 2D**) in the epithelial tissues of OLP. The location of IELs was marked (**Figure 2E**).

### ZO-1 and Occludin were expressed at low levels in OLP

**Figure 3:**
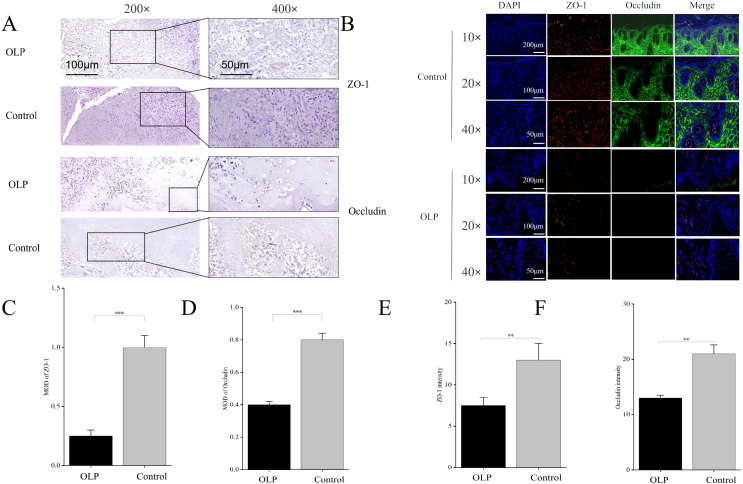
ZO-1 and Occludin were expressed at low levels in OLP. **(A)** ZO-1 and Occludin were predominantly localized in the main epithelial layer and universally distributed on the cell membrane. **(C, D)** The expression of ZO-1 and Occludin in the epithelial tissue of OLP was significantly downregulated compared to the control group, as detected by immunohistochemistry. **(B, E, F)** The expression of ZO-1 and Occludin in the epithelial tissue of OLP was significantly upregulated compared to the control group. *p < 0.05; **p < 0.01; ***p < 0.001. ZO-1, Zonula occludens protein 1; DAPI, 4’,6-Diamidino-2-phenylindole; OLP, Oral lichen planus; MOD, Mean optical density.

ZO-1 and Occludin were predominantly localized in the main epithelial layer (**Figure 3A**) and universally distributed on the cell membrane (**Figure 3A**). Quantitative MOD analysis demonstrated significantly reduced expression of ZO-1 and Occludin in the epithelial tissue of the OLP group compared to the control group (*p* < 0.001) (**Figures 3C, D**). Immunofluorescence staining results of ZO-1 and Occludin showed markedly downregulated expression in the epithelial tissue of OLP (*p* < 0.01) (**Figures 3B, E, F**).

### Within seven days, cell growth in the model showed an increasing trend, and the introduction of OLP IELs reduced the transepithelial resistance of KCs

**Figure 4:**
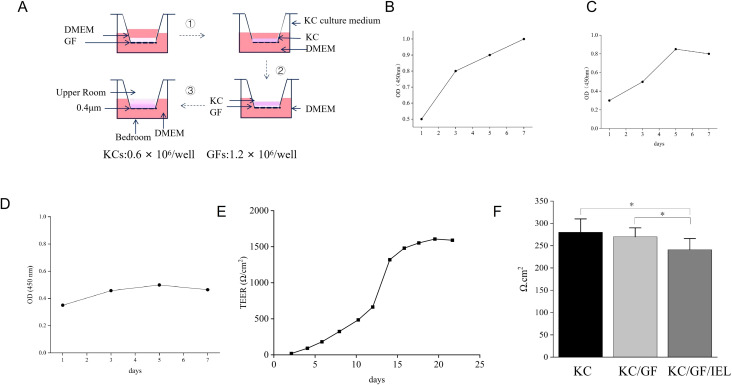
Construction of 3D simulation homing model of oral mucosal tissue and cell proliferation. **(A)** Schematic diagram of the construction of the 3D simulation oral mucosa tissue homing model. **(B)** GFs growth curve. **(C)** KCs growth curve. **(D)** Cells growth curve in homing model. **(E)** KCs were cultured in Transwell chambers for 21 days to form a firm monolayer of oral mucosal epithelial cells. **(F)** Transmembrane resistance: IELs reduced the transmembrane resistance of KCs. *p < 0.05. KC or KCs, Keratinocytes; GF or GFs, Gingival fibroblasts; IEL, Intraepithelial lymphocyte; DMEM, Dulbecco’s modified eagle medium; OD, Optical density; TEER, Trans-epithelial electrical resistance.

The construction process of the 3D simulation homing model of oral mucosal tissue was displayed (**Figure 4A**). On days 1, 3, 5, and 7, the OD values of GFs increased (**Figure 4B**), while the OD values of KCs (**Figure 4C**) and the model slightly decreased on day 7 (**Figure 4D**), indicating that the mucosal tissue structure began to form and cells gradually adhered to the transwell membrane. On the 10th day, full-thickness mucosal tissue was formed, and the TEER value was significantly increased. By the 21st day, a firm monolayer of KC epithelial cells was formed (**Figure 4E**).

Compared with the KC group, the transmembrane resistance in the KC/GF group showed no statistically significant difference. However, the KC/GF/IEL group exhibited a significant decrease in transmembrane resistance compared to both KC/GF group and KC group (*p* < 0.05) (**Figure 4F**), indicating that IELs compromised the integrity of KC cell membranes.

### E-cadherin and CD103 silencing inhibited proliferation and migration, and promoted apoptosis of KCs and OLP IELs

**Figure 5:**
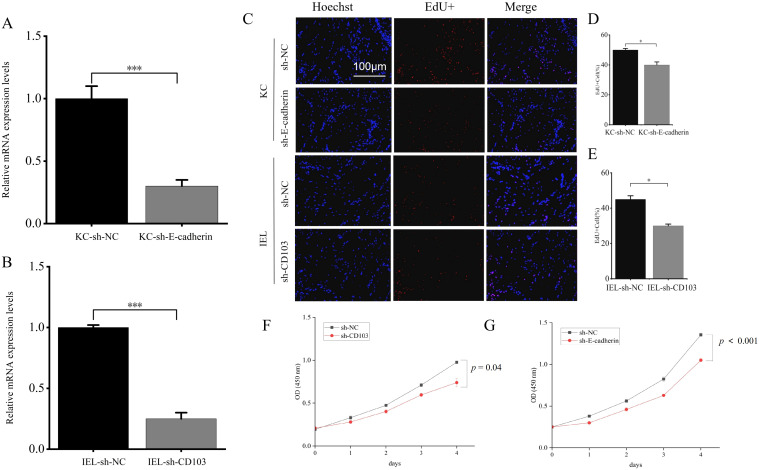
E-cadherin-silenced and CD103-silenced inhibited proliferation of KCs and OLP IELs. **(A)** The mRNA level of E-cadherin in KCs was significantly reduced in the sh-E-cadherin group compared to the sh-NC group. **(B)** The mRNA level of CD103 in IELs was significantly reduced in the sh-CD103 group compared to the sh-NC group. **(C–E)** Compared to the sh-NC group, the EdU-positive proportions of KCs in the sh-E-cadherin group and IELs in the sh-CD103 group were significantly reduced. **(F)** CD103 silencing leaded to decreased proliferation of IELs. **(G)** E-cadherin silencing leaded to decreased proliferation of KCs. *p < 0.05; **p < 0.01; ***p < 0.001. Scale bars 100 µm. KC, Keratinocytes; IEL, Intraepithelial lymphocyte; OD, Optical density; EdU, 5-Ethynyl-2’-deoxyuridine; NC, Negative control.

Stable infection with sh-E-cadherin lentiviral vector and sh-CD103 lentiviral vector was used to silence E-cadherin and CD103 expression in KCs and IELs, respectively, to investigate the effects of E-cadherin and CD103 silencing on the proliferation, migration, and apoptosis of KCs and IELs. RT-qPCR results confirmed that the mRNA level of E-cadherin in KCs was significantly reduced in the sh-E-cadherin group compared to the sh-NC group (*p* < 0.001) (**Figure 5A**); the mRNA level of CD103 in IELs was significantly reduced in the sh-CD103 group compared to the sh-NC group (*p* < 0.001) (**Figure 5B**). These results indicated that stable transfection silencing of E-cadherin and CD103 in KC and IEL cell lines was successfully constructed.

EdU staining results demonstrated that compared to the sh-NC group, the EdU-positive proportions of KCs in the sh-E-cadherin group and IELs in the sh-CD103 group were significantly reduced (*p* < 0.05) (**Figures 5C–E**). The CCK-8 assay also indicated that the proliferative activity of KCs in the silencing groups for E-cadherin was significantly decreased compared to the sh-NC group (*p* < 0.001) (**Figure 5G**). The proliferative activity of IELs in the silencing groups for CD103 was significantly decreased compared to the sh-NC group (*p* < 0.05) (**Figure 5F**). These results suggested that silencing of E-cadherin and CD103 can inhibit the proliferative capacity of KCs and IELs.

**Figure 6:**
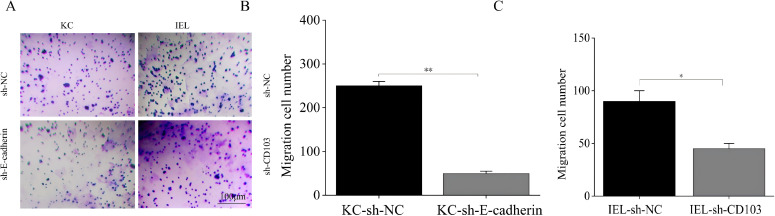
E-cadherin-silencing and CD103-silencing inhibited migration of KCs and OLP IELs. **(A, B)** Compared to the sh-NC group, the transmembrane migration capacity of KCs was significantly reduced in the sh-E-cadherin group. **(C)** The transmembrane migration capacity of IELs was significantly reduced in the sh-CD103 group (*p* < 0.05). *p < 0.05; **p < 0.01; ***p < 0.001. Scale bars 100 µm. KC, Keratinocytes; IEL, Intraepithelial lymphocyte; NC, Negative control.

After transwell-based *in vitro* seeding with different cell types and 24-hour incubation, compared to the sh-NC group, the transmembrane migration capacity of KCs was significantly reduced in the sh-E-cadherin group (*p* < 0.01) (**Figures 6A, B**), the transmembrane migration capacity of IELs was significantly reduced in the sh-CD103 group (*p* < 0.05) (**Figures 6A, C**). This suggested that silencing E-cadherin and CD103 inhibited the migratory capacity of KCs and IELs.

**Figure 7:**
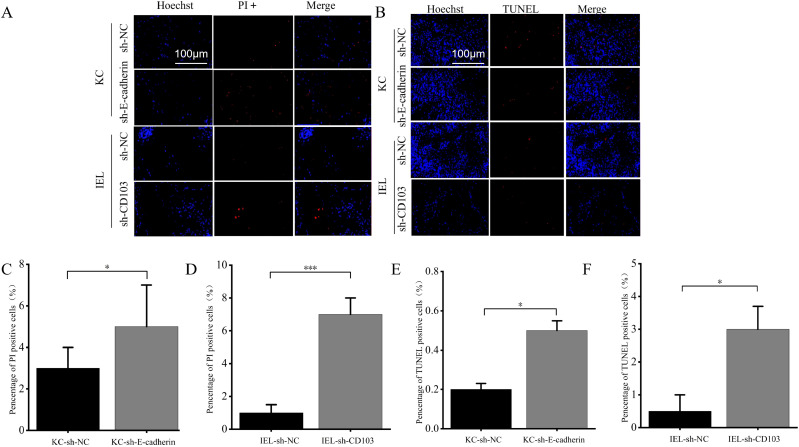
E-cadherin-silenced and CD103-silenced promoted apoptosis of KCs and OLP IELs. **(A)** PI staining. **(B)** TUNEL staining. **(C)** The E-cadherin silencing group had more PI-positive cells than sh-NC group (*p* < 0.05). **(D)** The CD103 silencing group had more PI-positive cells than sh-NC group (*p* < 0.001). **(E, F)** The E-cadherin and CD103 silencing group had more TUNEL-positive cells than sh-NC group (*p* < 0.05). *p < 0.05; **p < 0.01; ***p < 0.001. Scale bars 100 µm. KC, Keratinocytes; IEL, Intraepithelial lymphocyte; PI, Propidium iodide; TUNEL, Terminal deoxynucleotidyl transferase-mediated dUTP nick end labeling; NC, Negative control.

The E-cadherin silencing group had more PI-positive cells than sh-NC group (*p* < 0.05) (**Figures 7A, C**). The CD103 silencing group had more PI-positive cells than sh-NC group (*p* < 0.001) (**Figures 7A, D**). The E-cadherin and CD103 silencing group had more TUNEL-positive cells than sh-NC group (*p* < 0.05) (**Figures 7B, E, F**). These findings suggested that E-cadherin and CD103 silencing promoted apoptosis of KCs and IELs.

### E-cadehrin and CD103 silencing synergistically inhibited homing migration of OLP IELs

**Figure 8:**
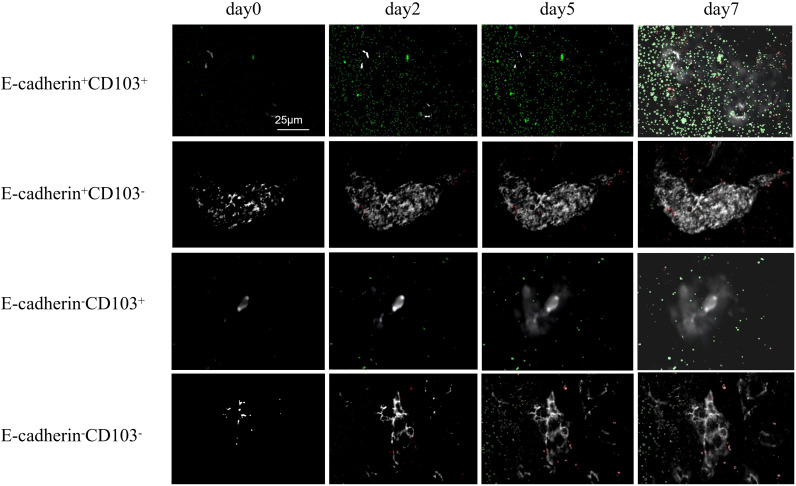
KCs-IELs co-cultures were monitored by confocal microscopy by SP5 in DIC from day 0 to day 7. E-cadherin^+^KC and E-cadherin^-^KC (RFP-labeled); CD103^+^IEL and CD103^-^IEL (GFP-labeled). Co-cultures were split before imaging. Scale bar 25 µm. Representative images of at least three independent experiments are shown. CD103, Integrin alpha-E; E-cadherin, Epithelial cadherin.

**Figure 9:**
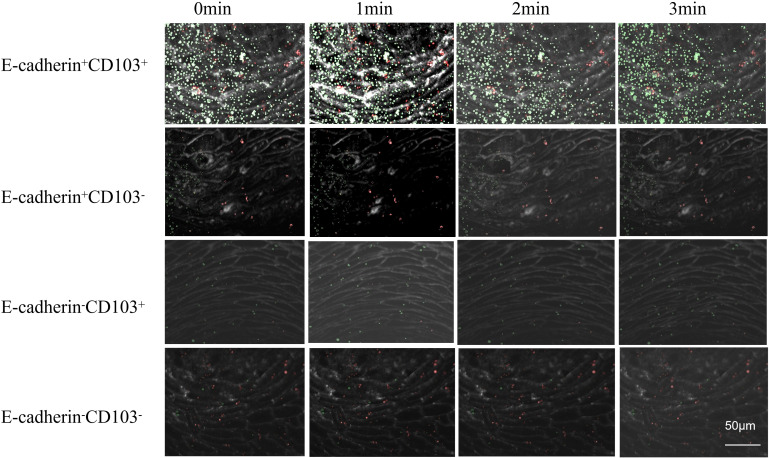
The mobility of IELs within the KCs layer can be monitored by live cell imaging and tracked with time-lapse studies. KC-IEL co-cultures were serially monitored by time-lapse imaging using spinning disc microscopy with 25× objective magnification in DIC. The time series demonstrates eight frames, with each frame taken 1 min apart. Scale bars 50 µm. CD103, Integrin alpha-E; E-cadherin, Epithelial cadherin.

**Figure 10:**
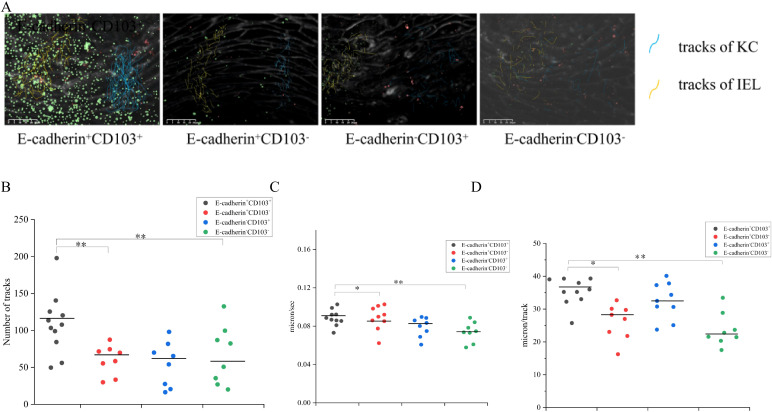
E-cadherin-silenced and CD103-silenced inhibited homing migration of OLP IELs. **(A)** Tracks of all IELs detectable during the observation period of 45 min are shown by yellow lines. Tracks of all KCs detectable during the observation period of 45 min are shown by blue lines. Scale bar 50 µm. Co-culture was incubated overnight before imaging. **(B)** The number of IEL tracks was quantified in KC-IEL co-cultures. A track is a contiguous, comprehensible movement of one cell over several frames and is represented by a single yellow line. **(C)** The mean speed of cells was calculated in KC-IEL co-cultures, respectively. **(D)** The maximum displacement, i.e., the mean of the highest 15 track length values per time-lapse movie. Co-cultures were incubated overnight before imaging. n = 8–10 per group. All data are representative of at least three independent experiments. * *p* < 0.05, ** *p* < 0.01; *** *p* < 0.001. KC, Keratinocytes; IEL, Intraepithelial lymphocyte; CD103, Integrin alpha-E; E-cadherin, Epithelial cadherin.

Through confocal microscopy combined with differential interference contrast (DIC) imaging technology (**Figure 8**), the localization and migration of IELs within the KC layer during the one-week co-culture period were clearly demonstrated. When live-cell imaging was performed using a rotating disk microscope and two-color imaging, it was observed that IELs underwent displacement over time (**Figure 9**). Compared to E-cadherin^+^CD103^+^, the number of tracks for E-cadherin^+^CD103^-^, E-cadherin^-^CD103^+^, and E-cadherin^-^CD103^-^ were significantly reduced (*p* < 0.01) (**Figures 10A, B**). Additionally, the speed and maximum displacement of E-cadherin^-^CD103^-^ were significantly lower than those of E-cadherin^+^CD103^+^ (*p* < 0.01) (**Figures 10A, C, D**), while the speed and maximum displacement of E-cadherin^+^CD103^-^ and E-cadherin^-^CD103^+^ were significantly lower than those of E-cadherin^+^CD103^+^ (*p* < 0.05) (**Figures 10A, C, D**).

### JAK2/STAT3 was highly expressed in OLP

**Figure 11:**
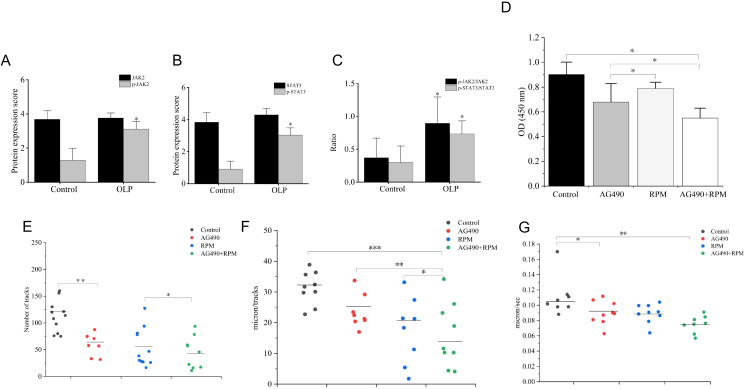
p-JAK2/JAK2 and p-STAT3/STAT3 were highly expressed in OLP. AG490 and RPM inhibited KCs proliferation. AG490 and RPM inhibited homing migration of OLP IELs. **(A–C)** p-JAK2/JAK2 and p-STAT3/STAT3 were significantly upregulated in OLP compared to controls (n = 6), as detected by western blot. **(D)** In the MTT assay, at 48 h, the OD450 values of the AG490 group, RPM group, and AG490+RPM group were significantly lower than those of the Control group. Compared with the AG490 group and RPM group, the OD450 values of the AG490+RPM group were significantly reduced. Additionally, the OD450 values of the AG490 group were significantly lower than those of the RPM group. **(E)** Compared with the control group, the AG490 group and RPM group exhibited a significant reduction in the number of migration tracks, while the AG490+RPM group showed a significantly reduced number of tracks. **(F, G)** Compared with the control group, the AG490 group and RPM group demonstrated a significant decrease in both speed and maximum displacement, and the AG490+RPM group exhibited a significant reduction in both speed and maximum displacement. *p < 0.05; **p < 0.01; ***p < 0.001. JAK2, Janus kinase 2; p-JAK2, Phosphorylated Janus kinase 2; STAT3, Signal transducer and activator of transcription 3; p-STAT3, Phosphorylated signal transducer and activator of transcription 3; OD, Optical density; AG490, (E)-N-Benzyl-2-cyano-3-(3,4-dihydroxyphenyl)prop-2-enamide; RPM, Ruxolitinib.

The expression of p-JAK2/JAK2 and p-STAT3/STAT3 were significantly upregulated in OLP, as detected by ELISA (*p* < 0.05) (n = 6) (**Figures 11A–C**).

### AG490 and RPM inhibited KCs proliferation

The MTT assay results demonstrated that at 48 h, the OD450 values of the AG490 group, RPM group, and AG490+RPM group were significantly lower than those of the control group (*p* < 0.05). Compared with the AG490 group and RPM group, the OD450 values of the AG490+RPM group were significantly reduced (*p* < 0.05). Additionally, the OD450 values of the AG490 group were significantly lower than those of the RPM group (*p* < 0.05) (**Figure 11D**).

### JAK/STAT inhibitors inhibited the homing migration of OLP IELs

Compared with the control group, the AG490 group and RPM group exhibited a significant reduction in the number of migration tracks (*p* < 0.01), while the AG490+RPM group showed a significantly reduced number of tracks (*p* < 0.001) (**Figure 11E**). Compared with the control group, the AG490 group and RPM group demonstrated a significant decrease in both speed and maximum displacement (*p* < 0.05), and the AG490+RPM group exhibited a significant reduction in both speed and maximum displacement (*p* < 0.001) (**Figures 11F, G**). These results suggest that dual inhibition of the keratinocyte JAK/STAT pathway exerts a synergistic inhibitory effect on IELs migration.

### Pro-apoptosis proteins Bax and caspase-3 were upregulated, anti-apoptosis Bcl-2 was downregulated

**Figure 12:**
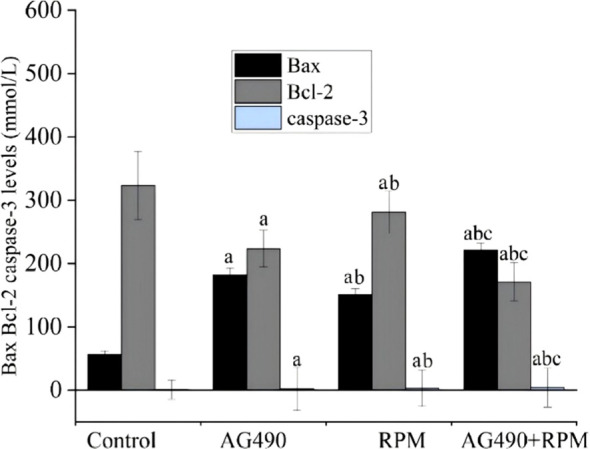
AG490 and RPM inhibited the expression of apoptosis-related proteins Bax and caspase-3 and promoted the expression of Bcl-2, as measured by ELISA. (a), compared with control, *p* < 0.05; (b), compared with AG490 group, *p* < 0.05; (c), compared with RPM group, *p* < 0.05. AG490, (E)-N-Benzyl-2-cyano-3-(3,4-dihydroxyphenyl)prop-2-enamide; RPM, Ruxolitinib; Bax, Bcl-2 associated X protein; Bcl-2, B-cell lymphoma/leukemia-2 gene; caspase-3, Cysteine-dependent aspartate-specific protease-3.

ELISA results demonstrated that compared with the control group, the protein expression levels of Bax and caspase-3 were significantly elevated in the AG490 group, RPM group, and AG490+RPM group, while the protein expression level of Bcl-2 was significantly reduced in all these groups (*p* < 0.05). Compared with the AG490 group and RPM group, the protein expression levels of Bax and caspase-3 were significantly elevated in the AG490+RPM group, and the protein expression level of Bcl-2 was significantly reduced in all these groups (*p* < 0.05). In the AG490 group, the protein expression levels of Bax and caspase-3 were significantly higher than those in the RPM group, while the protein expression level of Bcl-2 was significantly lower than that in the RPM group (*p* < 0.05) (**Figure 12**).

### The expression of JAK2/STAT3 pathway proteins was downregulated

**Figure 13:**
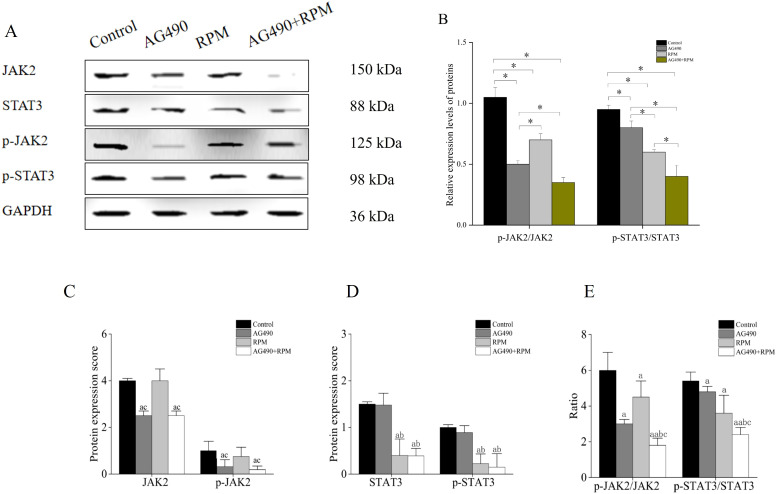
AG490 and RPM synergistically inhibited the phosphorylation of JAK2/STAT3. **(A, B)** AG490 and RPM selectively inhibited JAK2/STAT3 phosphorylation. *p < 0.05; **p < 0.01; ***p < 0.001. **(C)** JAK2 and p-JAK2 were downregulated in AG490 and AG490+RPM group. **(D)** STAT3 and p-STAT3 were downregulated in RPM and AG490+RPM group. **(E)** Compared with the control group, the protein ratios of p-JAK2/JAK2 and p-STAT3/STAT3 were significantly increased in the AG490 group, RPM group, and AG490+RPM group. Compared with the AG490 group and RPM group, the protein ratios of p-JAK2/JAK2 and p-STAT3/STAT3 were significantly decreased in the AG490+RPM group. a, compared with control, *p* < 0.05; b, compared with AG490 group, *p* < 0.05; c, compared with RPM group, *p* < 0.05; aa, compared with control, *p* < 0.01. AG490, (E)-N-Benzyl-2-cyano-3-(3,4-dihydroxyphenyl)prop-2-enamide; RPM, Ruxolitinib; JAK2, Janus kinase 2; p-JAK2, Phosphorylated Janus kinase 2; STAT3, Signal transducer and activator of transcription 3; p-STAT3: Phosphorylated signal transducer and activator of transcription 3; GAPDH, Glycerol-3-phosphate dehydrogenase.

Western blot results showed that compared with the control group, the p-JAK2/JAK2 and p-STAT3/STAT3 protein ratios were significantly increased in the AG490 group, RPM group, and AG490+RPM group (*p* < 0.05); compared with the AG490 group and RPM group, the p-JAK2/JAK2 and p-STAT3/STAT3 protein ratios were significantly decreased in the AG490+RPM group (*p* < 0.05); the p-JAK2/JAK2 protein ratio in the AG490 group was significantly lower than that in the RPM group (*p* < 0.05), while the p-STAT3/STAT3 protein ratio in the AG490 group was significantly higher than that in the RPM group (*p* < 0.05) (**Figures 13A, B**).

ELISA results showed that JAK2 and p-JAK2 were downregulated in AG490 and AG490+RPM group (*p* < 0.05) (**Figure 13C**). Compared with the control group, the protein ratios of p-JAK2/JAK2 and p-STAT3/STAT3 were significantly increased in the AG490 group and RPM group (*p* < 0.05) (**Figure 13E**), and AG490+RPM group (*p* < 0.01) (**Figure 13E**). STAT3 and p-STAT3 were downregulated in RPM and AG490+RPM group (*p* < 0.05) (**Figure 13D**). Compared with the AG490 group and RPM group, the protein ratios of p-JAK2/JAK2 and p-STAT3/STAT3 were significantly decreased in the AG490 + RPM group (*p* < 0.05) (**Figure 13E**).

### E-cadherin/CD103 reduced JAK2/STAT3 phosphorylation

**Figure 14:**
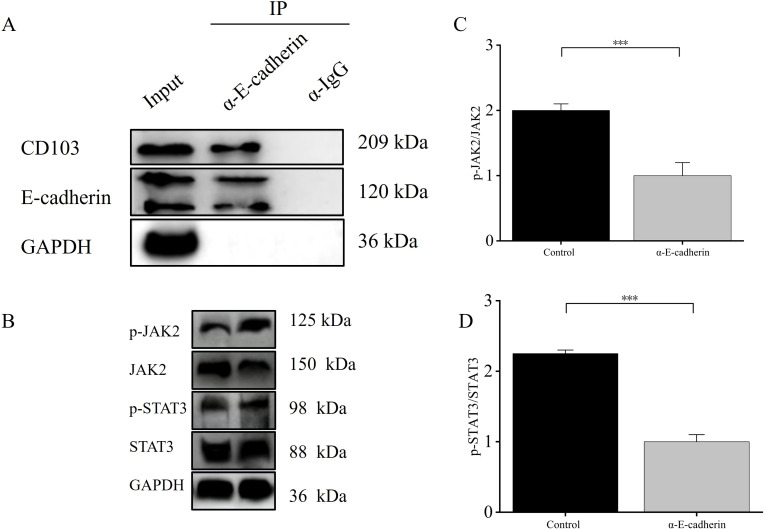
E-cadherin/CD103 reduced JAK2/STAT3 phosphorylation. **(A)** Co-immunoprecipitation of E-cadherin and CD103. **(B–D)** E-cadherin/CD103 downregulated the expression of p-JAK and p-STAT3 proteins. ***p < 0.001. IP, Immunoprecipitation; IgG, Immunoglobulin G; CD103, Integrin alpha-E; E-cadherin, Epithelial cadherin; JAK2, Janus kinase 2; p-JAK2, Phosphorylated Janus kinase 2; STAT3, Signal transducer and activator of transcription 3; p-STAT3, Phosphorylated signal transducer and activator of transcription 3; GAPDH, Glycerol-3-phosphate dehydrogenase.

Immunoprecipitation further confirmed the interaction between E-cadherin and CD103 (**Figure 14A**). Further examination of the expression changes of JAK2/STAT3 pathway-related proteins between the control group and α-E-cadherin group revealed that E-cadherin/CD103 could downregulate the expression of p-JAK and p-STAT3 proteins (*p* < 0.001) (**Figures 14B–D**).

### The expression of ZO-1 and Occludin mRNA was upregulated after using AG490 and RPM

**Figure 15:**
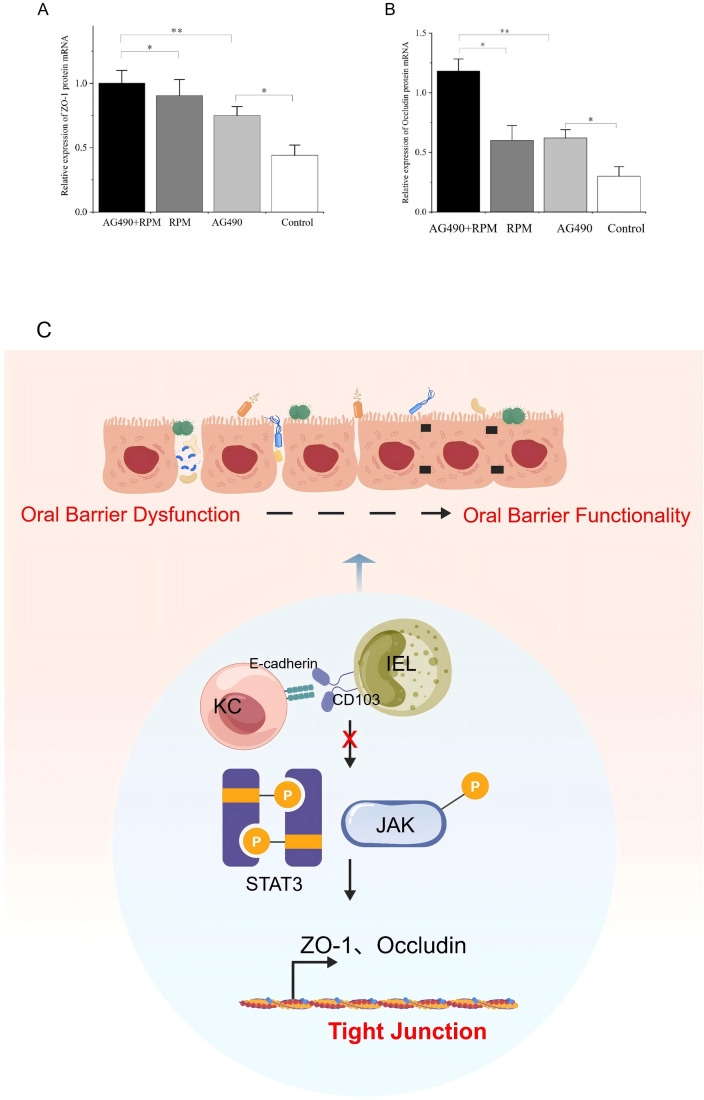
The expression of ZO-1 and Occludin mRNA was upregulated after using AG490 and RPM. **(A, B)** Compared with the control group, the expression of ZO-1 and Occludin mRNA increased in the AG490 group. The expression of ZO-1 and Occludin mRAN in the RPM group was upregulated. The expression of ZO-1 and Occludin mRNA was significantly increased in the AG490+RPM group. Compared with the RPM group, the AG490+RPM group showed increased expression of ZO-1 and Occludin mRNA. *p < 0.05; **p < 0.01; ***p < 0.001. **(C)** Schematic diagram of the regulatory network mechanism involving E-cadherin/CD103-JAK2/STAT3-ZO-1/Occludin in OLP. The binding of E-cadherin/CD103 downregulated JAK2/STAT3 phosphorylation in KCs and upregulated mucosal barrier molecules ZO-1 and Occludin, which helped maintain the integrity of the mucosal barrier. CD103, Integrin alpha-E; E-cadherin, Epithelial cadherin; JAK2, Janus kinase 2; STAT3, Signal transducer and activator of transcription 3; AG490, (E)-N-Benzyl-2-cyano-3-(3,4-dihydroxyphenyl)prop-2-enamide; RPM, Ruxolitinib; ZO-1, Zonula occludens protein 1; KC, Keratinocytes; IEL, Intraepithelial lymphocyte.

Compared with the control group, the expression of ZO-1 and Occludin mRNA increased in the AG490 group (*p* < 0.05) (**Figures 15A, B**). The expression of ZO-1 and Occludin mRAN in the RPM group was upregulated (*p* < 0.05) (**Figures 15A, B**). The expression of ZO-1 and Occludin mRNA was significantly increased in the AG490+RPM group (*p* < 0.001) (**Figures 15A, B**). Compared with the RPM group, the AG490+RPM group showed increased expression of ZO-1 and Occludin mRNA (*p* < 0.05) (**Figures 15A, B**). The above results suggested that the synergistic inhibitory effect of AG490 and RPM on JAK/STAT increased expression of mucosal barrier molecules. **Figure 15C** illustrates the regulatory network mechanism involving E-cadherin/CD103-JAK2/STAT3-ZO-1/Occludin in OLP.

## Discussion

IELs are a unique population of T cells that reside in the intestinal mucosal epithelium (27). The majority of IELs are T lymphocytes, with approximately 85% being CD8^+^ T cells and CD4^+^ T cells accounting for only about 5% (28). IELs can also interact with epithelial cells, secreting cytokines to regulate epithelial cell proliferation and maintain the mucosal barrier function of the intestinal epithelium (29). The present study was the first to investigate the molecular regulation mechanism of KCs on OLP IELs migration and homing. CD8α and CD103 expression was upregulated while E-cadherin expression was downregulated, and the binding between CD103 and E-cadherin was weakened. When CD103 and E-cadherin were silenced, the homing migration of OLP IELs was inhibited, indicating that the communication between OLP IELs and KCs depended on CD103 and E-cadherin. After inhibiting JAK2/STAT3, the phosphorylation level of JAK2/STAT3 decreased, and the expression of pro-apoptotic genes Bax, and caspase-3 was upregulated. Proliferative KCs were inhibited and apoptosis was induced, which would be beneficial for reducing abnormal epithelial hyperplasia in OLP. Meanwhile, upregulated expression of mucosal barrier molecules ZO-1 and Occludin will help maintain the integrity of the mucosal barrier.

ZO-1 and Occludin are commonly used as indicators to observe the barrier function and permeability of tight junctions in various tissues (30, 31). ZO-1 serves as a bridge between transmembrane proteins (such as Occludin) and the cytoskeletal protein actin. Occludin, the first transmembrane protein discovered, plays a crucial role in the assembly of tight junctions and the maintenance of their barrier function through its N-terminal domain. Its C-terminal domain is directly connected to ZO-1, and together they regulate structural changes in tight junctions. This characteristic is essential for the localization of Occludin between tight junctions and the regulation of intercellular permeability (32). We found that the expression of ZO-1 and Occludin in OLP was significantly reduced, indicating that the mucosal barrier integrity of OLP had been compromised.

GFs and KCs were selected to construct 3D simulation homing model of oral mucosal tissue to achieve the intuitiveness and controllability of cell culture. On the 7th day, OD values of cell growth in the model showed a slight decline, with KCs and GFs gradually adhering to the transwell membrane, indicating the initiation formation of the full-thickness mucosal tissue. LPS is a potent pro-inflammatory factor with immunological and cytological activities that can induce inflammatory responses. It also exhibits strong antigenicity by activating T cells and B cells, thereby mobilizing cellular immune responses. OLP is primarily mediated by T cells; therefore, LPS stimulation is selected to simulate the inflammatory microenvironment of OLP. LPS stimulation on KCs resulted in a significant reduction in transmembrane resistance in the KC/GF/IEL group, suggesting that IELs may be involved in destruction of the integrity of KCs membranes. This result preliminarily confirmed the communication between IELs and KCs.

Therefore, it is essential to determine the roles of E-cadherin and CD103 in the communication between KCs and IELs. Previous studies have found that the vast majority of basal KCs in OLP exhibit focal loss of E-cadherin (33). Meanwhile, E-cadherin was also hypermethylated in OLP (34). In our study, E-cadherin silencing inhibited KCs proliferation and migration and promoted KCs apoptosis; meanwhile, CD103 silencing inhibited IELs proliferation and migration, and promoted IELs apoptosis. Live-cell imaging and time-lapse imaging techniques confirmed that the migration tracks and speed of IELs were CD103-dependent. When E-cadherin was silenced, communication between KCs and IELs had noticeably decreased. These results suggested that the interaction between CD103 and E-cadherin guided OLP IELs to efficiently and rapidly cross the mucosal epithelial layer.

The WHO has designated OLP as oral potentially malignant disorders (3). The activation of the JAK/STAT signaling pathway is precisely mediated by phosphorylation activities, which are an essential post-translational modification of the STAT family proteins. Through its persistent activation, the JAK/STAT pathway, a crucial intracellular signaling axis, has been shown to be implicated in a number of immune system diseases (25). In comparison to control, we found that OLP had much greater JAK2/STAT3 expression. The JAK2 inhibitor AG490 and the STAT3 pathway inhibitor RPM both successfully inhibited KCs proliferation; when the two inhibitors were combined, synergistic inhibition effects were observed. These results suggested that, in contrast to single-target therapies, simultaneous inhibition of the JAK/STAT signaling cascade at many upstream and downstream nodes may constitute a more successful therapeutic approach for OLP.

OLP has been effectively treated with tofacitinib, a JAK1/3 inhibitor (23, 24). Due to its ability to disrupt IFN-γ signaling via the JAK2/STAT1 pathway, baricitinib may also be a viable treatment option for OLP (35). The significance and viability of the JAK/STAT pathway as a therapeutic target for OLP were supported by these investigations as well as our findings. After receiving AG490 and RPM, we observed a significant decrease in the protein levels of p-JAK2 and p-STAT3, indicating that the medications successfully prevented the JAK2/STAT3 signaling pathway from being activated. A crucial stage in the activation of the JAK and STAT proteins is phosphorylation. By directly lowering the phosphorylation level of its downstream substrate STAT3, p-JAK2 inhibition prevents the entire signaling pathway from cascading. OLP exhibited high expression of apoptosis-related proteins, including P21, Bax, and caspase-3 (36). Our results showed that inhibitor treatment markedly increased the expression of the pro-apoptotic gene Bax while decreasing the expression of the anti-apoptotic gene Bcl-2, resulting in a higher ratio of Bax to Bcl-2. This ultimately started the apoptosis program by activating the downstream effector caspase-3. We observed aberrant activation of the JAK/STAT signaling pathway in OLP, despite the fact that this finding might appear to promote OLP progression. As a result, JAK2/STAT3 inhibition encouraged KCs to differentiate and undergo apoptosis normally while reducing their excessive proliferation. This aligned with current research findings (37). On the other hand, JAK/STAT pathway inhibition also reduced T-cell-directed anti-keratinocyte reactions, which had therapeutic benefits for OLP (38).

According to our research, the combined use of AG490 and RPM performed better than monotherapy across the board. There could be two components to the underlying mechanisms. First, simultaneously inhibiting the downstream core transcription factor STAT3 and the upstream kinase JAK2 can more completely block the signaling cascade from the standpoint of a vertical signaling pathway, preventing compensatory or bypass activation that could happen with single-target inhibition. Second, it is important to remember that RPM has a traditional role as an NF-κB inhibitor, even though it is utilized in this study as an inhibitor of the STAT3 pathway. Through intricate mutual regulatory networks, the JAK/STAT and NF-κB pathways were linked (39, 40). Consequently, there may be a synergistic effect when AG490 and RPM are combined to inhibit two crucial pathways that are necessary for KCs development. As the JAK/STAT pathway was inhibited, our results verified that ZO-1 and Occludin expression rose, suggesting increased tight junction protein expression.

In conclusion, this study suggested that the regulation of KCs on OLP IELs homing and migration depended on the E-cadherin of KCs and CD103 on OLP IELs. By downregulating JAK2/STAT3 phosphorylation in KCs, KCs proliferation can be inhibited and apoptosis induced, which would be beneficial for the epithelial dysplasia of OLP. Meanwhile, upregulated expression of mucosal barrier molecules will help maintain the integrity of the mucosal barrier.

## Data Availability

The original contributions presented in the study are included in the article/supplementary material. Further inquiries can be directed to the corresponding author.

## References

[1] Feldmeyer L Suter VG Oeschger C Cazzaniga S Bornstein MM Simon D . Oral lichen planus and oral lichenoid lesions - an analysis of clinical and histopathological features. J Eur Acad Dermatol Venereology: Jeadv. (2020) 34:e104–7. doi: 10.1111/jdv.15981. PMID: 31568593

[2] Aguirre-Urizar JM Alberdi-Navarro J Lafuente-Ibáñez de Mendoza I Marichalar-Mendia X Martínez-Revilla B Parra-Pérez C . Clinicopathological and prognostic characterization of oral lichenoid disease and its main subtypes: a series of 384 cases. Med Oral Patologia Oral Y Cbucal. (2020) 25:e554–62. doi: 10.4317/medoral.23576. PMID: 32388519 PMC7338060

[3] Warnakulasuriya S . Oral potentially Malignant disorders: a comprehensive review on clinical aspects and management. Oral Oncol. (2020) 102:104550. doi: 10.1016/j.oraloncology.2019.104550. PMID: 31981993

[4] El-Howati A Thornhill MH Colley HE Murdoch C . Immune mechanisms in oral lichen planus. Oral Dis. (2023) 29:1400–15. doi: 10.1111/odi.14142. PMID: 35092132

[5] Qing M Yang D Shang Q Peng J Deng J Lu J . CD8(+) tissue-resident memory T cells induce oral lichen planus erosion via cytokine network. Elife. (2023) 12:e83981. doi: 10.7554/eLife.83981. PMID: 37555396 PMC10465124

[6] Society of Oral Medicine Chinese Stomatological Association . Guideline for the diagnosis and treatment of oral lichen planus (revision). Zhonghua Kou Qiang Yi Xue Za Zhi. (2022) 57:115–21. doi: 10.3760/cma.j.cn112144-20211115-00505. PMID: 35152645

[7] Cheng YS Gould A Kurago Z Fantasia J Muller S . Diagnosis of oral lichen planus: a position paper of the American Academy of Oral and Maxillofacial Pathology. Oral Surgery Oral Medicine Oral Pathol And Oral Radiol. (2016) 122:332–54. doi: 10.1016/j.oooo.2016.05.004. PMID: 27401683

[8] Carrozzo M Porter S Mercadante V Fedele S . Oral lichen planus: a disease or a spectrum of tissue reactions? Types, causes, diagnostic algorhythms, prognosis, management strategies. Periodontology 2000. (2019) 80:105–25. doi: 10.1111/prd.12260. PMID: 31090143

[9] Liu Y Liu G Liu Q Tan J Hu X Wang J . The cellular character of liquefaction degeneration in oral lichen planus and the role of interferon gamma. J Oral Pathol Med. (2017) 46:1015–22. doi: 10.1111/jop.12595. PMID: 28556960

[10] Bao CF Wang F Zhou DY Zhou G . CD4(+)CD8αα(+) is the dominant phenotype of intraepithelial lymphocytes and regulated by ThPOK and Runx3 in oral lichen planus. J Oral Pathol Med. (2024) 53:480–90. doi: 10.1111/jop.13564. PMID: 38866540

[11] Ley K Rivera-Nieves J Sandborn WJ Shattil S . Integrin-based therapeutics: biological basis, clinical use and new drugs. Nat Rev Drug Discov. (2016) 15:173–83. doi: 10.1038/nrd.2015.10. PMID: 26822833 PMC4890615

[12] Vermeire S O'Byrne S Keir M Williams M Lu TT Mansfield JC . Etrolizumab as induction therapy for ulcerative colitis: a randomised, controlled, phase 2 trial. Lancet (London England). (2014) 384:309–18. doi: 10.1016/S0140-6736(14)60661-9. PMID: 24814090

[13] Rivera-Nieves J Gorfu G Ley K . Leukocyte adhesion molecules in animal models of inflammatory bowel disease. Inflammatory Bowel Dis. (2008) 14:1715–35. doi: 10.1002/ibd.20501. PMID: 18523998 PMC2733908

[14] Rübsam M Broussard JA Wickström SA Nekrasova O Green KJ Niessen CM . Adherens junctions and desmosomes coordinate mechanics and signaling to orchestrate tissue morphogenesis and function: an evolutionary perspective. Cold Spring Harbor Perspect Biol. (2018) 10:a029207. doi: 10.1101/cshperspect.a029207. PMID: 28893859 PMC6211388

[15] Hardenberg JB Braun A Sch N MP . A Yin and Yang in epithelial immunology: the roles of the α(E)(CD103)β(7) integrin in T cells. J Invest Dermatol. (2018) 138:23–31. doi: 10.1016/j.jid.2017.05.026. PMID: 28941625

[16] Zundler S Schillinger D Fischer A Atreya R López-Posadas R Watson A . Blockade of αEβ7 integrin suppresses accumulation of CD8(+) and Th9 lymphocytes from patients with IBD in the inflamed gut *in vivo*. Gut. (2017) 66:1936–48. doi: 10.1136/gutjnl-2016-312439. PMID: 27543429

[17] Lockhart A Mucida D Bilate AM . Intraepithelial lymphocytes of the intestine. Annu Rev Immunol. (2024) 42:289–316. doi: 10.1146/annurev-immunol-090222-100246. PMID: 38277691 PMC11608099

[18] Song X Ma J . SRRM1 promotes the proliferation, migration, and invasion of hepatocellular carcinoma cells by regulating the JAK/STAT signaling pathway. Tissue Cell. (2022) 79:101954. doi: 10.1016/j.tice.2022.101954. PMID: 36270072

[19] Mansur F Arshad T Liska V Manzoor S . Interleukin-22 promotes the proliferation and migration of hepatocellular carcinoma cells via the phosphoinositide 3-kinase (PI3K/AKT) signaling pathway. Mol Biol Rep. (2023) 50:5957–67. doi: 10.1007/s11033-023-08542-x. PMID: 37264148

[20] Al-Rawashde FA Al-Wajeeh AS Vishkaei MN Saad HKM Johan MF Taib WRW . Thymoquinone inhibits JAK/STAT and PI3K/Akt/ mTOR signaling pathways in MV4–11 and K562 myeloid leukemia cells. Pharm (Basel Switzerland). (2022) 15:1123. doi: 10.3390/ph15091123. PMID: 36145344 PMC9504933

[21] Alves de Medeiros AK Speeckaert R Desmet E Van Gele M De Schepper S Lambert J . JAK3 as an emerging target for topical treatment of inflammatory skin diseases. PloS One. (2016) 11:e0164080. doi: 10.1371/journal.pone.0164080. PMID: 27711196 PMC5053514

[22] Abduelmula A Bagit A Mufti A Yeung KCY Yeung J . The use of Janus kinase inhibitors for lichen planus: an evidence-based review. J Cutaneous Med And Surg. (2023) 27:271–6. doi: 10.1177/12034754231156100. PMID: 36815857 PMC10291104

[23] Yang CC Khanna T Sallee B Christiano AM Bordone LA . Tofacitinib for the treatment of lichen planopilaris: a case series. Dermatologic Ther. (2018) 31:e12656. doi: 10.1111/dth.12656. PMID: 30264512 PMC6585740

[24] Damsky W Wang A Olamiju B Peterson D Galan A King B . Treatment of severe lichen planus with the JAK inhibitor tofacitinib. J Allergy And Clin Immunol. (2020) 145:1708–10.e2. doi: 10.1111/dth.12656. PMID: 32018031

[25] Djidjik R Lamara Mahammed L Berkani LM Allam I Benmoussa AH Gharnaout M . JAK/STAT in human diseases: a common axis in immunodeficiencies and hematological disorders. Front Immunol. (2025) 16:1669688. doi: 10.3389/fimmu.2025.1669688. PMID: 41438753 PMC12719510

[26] Wang F Zhang J Zhou G . The mTOR-glycolytic pathway promotes T-cell immunobiology in oral lichen planus. Immunobiology. (2020) 225:151933. doi: 10.1016/j.imbio.2020.151933. PMID: 32201095

[27] Binda E Erhart D Schenk M Zufferey C Renzulli P Mueller C . Quantitative isolation of mouse and human intestinal intraepithelial lymphocytes by elutriation centrifugation. J Immunol Methods. (2009) 344:26–34. doi: 10.1016/j.jim.2009.02.006. PMID: 19278662

[28] Yang H Gumucio DL Teitelbaum DH . Intestinal specific overexpression of interleukin-7 attenuates the alternation of intestinal intraepithelial lymphocytes after total parenteral nutrition administration. Ann Surg. (2008) 248:849–56. doi: 10.1097/SLA.0b013e31818a1522. PMID: 18948814 PMC2597497

[29] Rei Ig S Hackenbruch C Velmeyer NH . Isolation of T cells from the gut. Methods Mol Biol (Clifton NJ). (2014) 1193:21–5. doi: 10.1007/978-1-4939-1212-4_3. PMID: 25150993

[30] Apodaca G Kiss S Ruiz W Meyers S Zeidel M Birder L . Disruption of bladder epithelium barrier function after spinal cord injury. Am J Physiol Renal Physiol. (2003) 284:F966–76. doi: 10.1152/ajprenal.00359.2002. PMID: 12527557

[31] Dörfel MJ Westphal JK Bellmann C Krug SM Cording J Mittag S . CK2-dependent phosphorylation of occludin regulates the interaction with ZO-proteins and tight junction integrity. Cell Communication And Signaling: Ccs. (2013) 11:40. doi: 10.1186/1478-811X-11-40. PMID: 23758859 PMC3695765

[32] Kawedia JD Jiang M Kulkarni A Waechter HE Matlin KS Pauletti GM . The protein kinase A pathway contributes to Hg2^+^-induced alterations in phosphorylation and subcellular distribution of occludin associated with increased tight junction permeability of salivary epithelial cell monolayers. J Pharmacol Exp Ther. (2008) 326:829–37. doi: 10.1124/jpet.107.135798. PMID: 18550693 PMC2677297

[33] Bar JK Cierpikowski P Lis-Nawara A Duc P Hałoń A Radwan-Oczko M . Comparison of p53, HSP90, E-cadherin and HPV in oral lichen planus and oral squamous cell carcinoma. Acta Otorhinolaryngol Ital. (2021) 41:514–22. doi: 10.14639/0392-100X-N1450. PMID: 34928263 PMC8686798

[34] Chujo T Yoshida K Takai R Uehara O Matsuoka H Morikawa T . Analysis of DNA methylation of E-cadherin and p16(ink4a) in oral lichen planus/oral lichenoid lesions. Clin And Exp Dental Res. (2021) 7:205–10. doi: 10.1002/cre2.355. PMID: 33274608 PMC8019760

[35] Shao S Tsoi LC Sarkar MK Xing X Xue K Uppala R . IFN-γ enhances cell-mediated cytotoxicity against keratinocytes via JAK2/STAT1 in lichen planus. Sci Transl Med. (2019) 11:eaav7561. doi: 10.1126/scitranslmed.aav7561. PMID: 31554739 PMC7285657

[36] Bascones C Gonzalez-Moles MA Esparza G Bravo M Acevedo A Gil-Montoya JA . Apoptosis and cell cycle arrest in oral lichen planus hypothesis on their possible influence on its Malignant transformation. Arch Oral Biol. (2005) 50:873–81. doi: 10.1016/j.archoralbio.2005.02.005. PMID: 16137496

[37] Yu YJ Xu YY Lan XO Liu XY Zhang XL Gao XH . Shikonin induces apoptosis and suppresses growth in keratinocytes via CEBP-δ upregulation. Int Immunopharmacol. (2019) 72:511–21. doi: 10.1016/j.intimp.2019.04.047. PMID: 31075711

[38] Xu H Zhang X Wang X Li B Yu H Quan Y . Cellular spermine targets JAK signaling to restrain cytokine-mediated autoimmunity. Immunity. (2024) 57:1796–811.e8. doi: 10.1016/j.immuni.2024.05.025. PMID: 38908373

[39] Wang J Luo H Yang L Li Y . Baicalein induces apoptosis and reduces inflammation in LPS-stimulated keratinocytes by blocking the activation of NF-κB: implications for alleviating oral lichen planus. Cell And Mol Biol (Noisy-Le-Grand France). (2016) 62:55–60 27453273

[40] Yoon CS Lee H Liu Z Dong L Lee G Kim N . Cycloolivil isolated from Nardostachys jatamansi inhibits TNF-α/IFN-γ-induced chemokine production by blocking NF-κB and JAK/STAT activation in HaCaT keratinocytes. Int J Mol Sci. (2024) 25:3342. doi: 10.3390/ijms25063342. PMID: 38542316 PMC10969846

